# Perioperative Protocol of Ankle Fracture and Distal Radius Fracture Based on Enhanced Recovery after Surgery Program: A Multicenter Prospective Clinical Controlled study

**DOI:** 10.1155/2022/3458056

**Published:** 2022-06-07

**Authors:** Ting Li, Zhi-Jian Sun, Yan Zhou, Wei-Tong Sun, Peng-Cheng Wang, Xin-Yu Cai, Jun-Bo Liang, Jing-Ming Dong, Da-Peng Zhou, Kai Yu, Ming-Xin Wu, Jiu-Sheng He, Liang-Yuan Wen, Bao-Qing Yu, Jian Wang, Jun Yang, Feng-Fei Lin, Bing-Zuan Li, Zong-Xin Shi, Bao-Jun Wang, Ai-Guo Wang, Gui-Ling Peng, Xu Sun, Hong-Hao Xiao, Meng Mi, Xia Zhao, Chang-Run Li, Gang Liu, Shao-Liang Li, Hang-Yu Gu, Yuan Zhou, Zhe-Lun Tan, Xin-Bao Wu

**Affiliations:** ^1^Department of Orthopaedic Trauma, Beijing Jishuitan Hospital, Beijing, China; ^2^Department of Anesthesiology, Beijing Jishuitan Hospital, Beijing, China; ^3^Department of Orthopaedic Trauma, The Third Hospital of Hebei Medical University, Shijiazhuang, China; ^4^Department of Orthopaedics, Shanghai Tenth People's Hospital, Shanghai, China; ^5^Department of Orthopaedics, Taizhou Hospital of Zhejiang Province, Taizhou, China; ^6^Department of Orthopaedics, Tianjin Hospital, Tianjin, China; ^7^Department of Orthopaedics, The General Hospital of Shenyang Military Command, Shenyang, China; ^8^Department of Orthopaedics, Peking University BinHai Hospital, Tianjin, China; ^9^Department of Orthopaedics, Huizhou Third People's Hospital, Huizhou, China; ^10^Department of Orthopaedics, Beijing Shunyi District Hospital, Beijing, China; ^11^Department of Orthopaedics, Beijing Hospital, Beijing, China; ^12^Department of Orthopaedics, Shanghai Pudong Hospital, Fudan University Pudong Medical Center, Shanghai, China; ^13^Department of Orthopaedics, The First Hosptial of Fangshan District, Beijing, China; ^14^Department of Orthopaedics, Shengjing Hospital, China Medical University, Shenyang, China; ^15^Department of Orthopaedics, The Second Hospital of Fuzhou, Fuzhou, China; ^16^Department of Orthopaedics, Quanzhou Orthopaedic Traumatological Hospital, Quanzhou, China; ^17^Department of Orthopaedics, Liangxiang Teaching Hospital, Capital Medical University, Beijing, China; ^18^Department of Orthopaedics, Beijing Friendship Hospital, Capital Medical University, Beijing, China; ^19^Department of Orthopaedics, Zhengzhou Orthopaedics Hospital, Zhengzhou, China; ^20^Department of Nurtrition, Beijing Jishuitan Hospital, Beijing, China

## Abstract

**Background:**

The enhanced recovery after surgery (ERAS) program is aimed to shorten patients' recovery process and improve clinical outcomes. This study aimed to compare the outcomes between the ERAS program and the traditional pathway among patients with ankle fracture and distal radius fracture.

**Methods:**

This is a multicenter prospective clinical controlled study consisting of 323 consecutive adults with ankle fracture from 12 centers and 323 consecutive adults with distal radial fracture from 13 centers scheduled for open reduction and internal fixation between January 2017 and December 2018. According to the perioperative protocol, patients were divided into two groups: the ERAS group and the traditional group. The primary outcome was the patients' satisfaction of the whole treatment on discharge and at 6 months postoperatively. The secondary outcomes include delapsed time between admission and surgery, length of hospital stay, postoperative complications, functional score, and the MOS item short form health survey-36.

**Results:**

Data describing 772 patients with ankle fracture and 658 patients with distal radius fracture were collected, of which 323 patients with ankle fracture and 323 patients with distal radial fracture were included for analysis. The patients in the ERAS group showed higher satisfaction levels on discharge and at 6 months postoperatively than in the traditional group (*P* < 0.001). In the subgroup analysis, patients with distal radial fracture in the ERAS group were more satisfied with the treatment (*P*=0.001). Furthermore, patients with ankle fracture had less time in bed (*P* < 0.001) and shorter hospital stay (*P* < 0.001) and patients with distal radial fracture received surgery quickly after being admitted into the ward in the ERAS group than in the traditional group (*P*=0.001).

**Conclusions:**

Perioperative protocol based on the ERAS program was associated with high satisfaction levels, less time in bed, and short hospital stay without increased complication rate and decreased functional outcomes.

## 1. Introduction

Enhanced recovery after surgery (ERAS) is a multimodal, multidisciplinary approach for the care of the surgical patient based on published evidence, aiming to reduce physical and psychological stress and achieve rapid recovery [1]. This concept originated from gastrointestinal surgery in the late 20^th^ century. Previous studies have shown that the implementation of the ERAS clinical pathway shortens the length of hospital stay and reduces the medical costs, complications, and readmission, while satisfaction and safety after discharge are increased [2–4]. Due to the excellent clinical results, ERAS has rapidly adapted by other surgical fields, and several ERAS guidelines for gastrointestinal surgery, hepatobiliary surgery, urology, and obstetrics and gynecology have been introduced [5–7]. Nonetheless, ERAS started late in orthopedic trauma, and thus, the only well-developed ERAS pathway is for elderly hip fractures. A standard ERAS protocol for hip fractures in the elderly has been established in England; several studies have reported markedly shortened length of hospital stay and complications among these patients [8, 9]. However, the perioperative protocol based on the ERAS program in managing other common fractures is still being explored.

Ankle and distal radial fractures are the two common fractures managed by orthopedic surgeons. Although indications for surgical intervention for the above two fracture types are well defined, the standard perioperative protocol based on the ERAS program remains unclear. Based on the updated ERAS guidelines of other operations, the perioperative pathways for ankle and distal radial fractures have been optimized in China, focusing on short perioperative fasting duration, minimally invasive approaches, minimizing drains and tubes, multimodal pain control, and early mobilization.

In this study, we compared the process and outcome measures between the ERAS and traditional pathways among the two target populations: patients with ankle fracture and distal radial fracture undergoing open reduction and internal fixation. Thus, we determined the effectiveness of the ERAS program.

## 2. Methods

### 2.1. Study Design and Participants

This study was a prospective, multicenter clinical controlled study approved by the ethics committee of Beijing Jishuitan Hospital, Beijing, China (JST-201707-10, JST-201707-11). Written informed consent was obtained from patients before participation in the study. No financial compensation was provided to the participants. All centers partook voluntarily, and only centers with valid cases >5 were finally analyzed. Patients with a primary diagnosis of ankle fracture and distal radial fracture were enrolled between January 2017 and December 2018 in the centers included in this study. All patients were treated with open reduction and internal fixation. The inclusion criteria were as follows: (1) age between 16 and 70 years; (2) primary diagnosis of ankle fracture or distal radial fracture (combined with ulnar styloid fracture or distal ulnar fracture); (3) no serious systemic disease with a preoperative American Society of Anesthesiologists (ASA) classification I-II; (4) voluntary participation with informed consent. The exclusion criteria were as follows: (1) combined with other fractures; (2) old ankle fractures and distal radial fractures; (2) open fractures (excluding Gustilo type I); (3) diagnosis of diabetes mellitus or other severe metabolic diseases; (4) diagnosis of gastric emptying disorders; (5) alcohol dependence or history of drug abuse; (6) breastfeeding or pregnant women; (7) history of allergy to multiple drugs. The centers were considered ERAS centers if they had a multidisciplinary ERAS team and implemented the protocol described in this article. Data were collected at each center by a trained surgeon and deidentified before entry into an internet-based electronic case record form designed specifically for this study.

### 2.2. ERAS Pathway

The ERAS pathway in this study was a standard perioperative protocol focusing mainly on pain management, perioperative fasting, and avoiding drains and urinary tubes. For pain management, we prescribed oral multimodal analgesics preoperatively and opioid-free analgesia based on nerve block postoperatively; a standard anesthesia protocol was followed for a painless procedure. For perioperative fasting, we minimized the fasting time as much as possible with low risks of pulmonary aspiration to reduce perioperative stress. For tubes management, urinary catheterization rate was reduced and meticulous hemostasis was achieved intraoperatively to avoid wound drains. The basic components of the multidisciplinary and multimodal ERAS pathway are listed in Table 1.

### 2.3. Process of Care Metrics

We evaluated three ERAS perioperative care elements using the following process of care metrics. For perioperative fasting management, we evaluated the perioperative fasting period for solids and liquids. For perioperative time management, the elapsed time between admission and surgery, the elapsed time between injury and surgery, the length of operation, and the length of hospital stay were evaluated. For perioperative nonsurgical management, we assessed the use of regional anesthesia, nonsteroidal anti-inflammatory drugs (NSAIDS), patient-controlled analgesia (PCA), antiemetic, urinary catheterization, and wound drains.

### 2.4. Outcome Metrics

Our primary outcome was the patients' satisfaction level of the whole treatment on discharge and at 6 months postoperatively. We divided the satisfaction into four levels A–D: A is the highest and D is dissatisfaction. The secondary outcomes include delapsed time between admission and surgery, elapsed time between injury and surgery, length of hospital stay, postoperative complications, and functional evaluation. Postoperative complications included loss of reduction, screw breakage by routine evaluation of postoperative radiographs, wound infection, deep vein thrombosis, and so on. American Orthopedic Foot and Ankle Society (AOFAS) ankle hindfoot scale and patient-related wrist evaluation (PRWE) were used to evaluate the postoperative function of the injured ankle and wrist, respectively, at 6 months after surgery. For patients with ankle fracture, the postoperative time in bed was also recorded as the secondary outcome.

### 2.5. Statistical Analysis

Statistical analysis was performed using IBM Statistical Package for Social Sciences Statistics (Version 22.0, IBM, Elmonck, New York, USA). All quantitative data were tested for normality using the Shapiro–Wilk test. The discrete variables were described as number and percentage, and the differences were compared using Fisher's exact or Pearson test. The continuous data were expressed as median (interquartile range (IQR)) and analyzed using the Wilcoxon rank-sum test. Multiple logistic regression analysis was conducted using patient demographic, injury, and perioperative management characteristics to identify the independent risk factors to assess the satisfaction level of the patients on discharge. *P* < 0.05 indicated statistical significance.

## 3. Results

### 3.1. Demographic Characteristics

**Figure 1:**
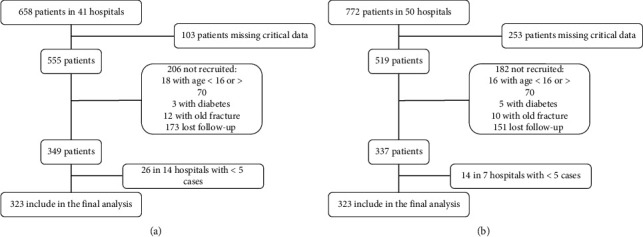
Schematic of included patients.

The data of 772 patients with ankle fracture in 50 hospitals and 658 patients with distal radial fracture in 41 hospitals were collected (Figure 1). Of these, 323 patients with ankle fracture in 12 hospitals and 323 patients with distal radial fracture in 13 hospitals were included for analysis. According to the centers where the surgery was performed, 181 (56.0%) patients with ankle fracture and 104 (32.2%) patients with distal radial fracture were treated using the ERAS perioperative protocol. The demographic characteristics of the included patients are shown in Table 2.

### 3.2. ERAS Process

Most process metrics of the included patients demonstrated significant differences between the ERAS and the traditional groups. For perioperative fasting management, no significant difference was observed between the two groups in the perioperative fasting period for solids, but that for preoperative liquids (6.5 h *vs*. 13.8 h, *P* < 0.001) and postoperative solids (3.0 h *vs*. 5.5 h, *P* < 0.001) and liquids (1.5 h *vs*. 4.0 h, *P* < 0.001) of the ERAS group was significantly shorter than the traditional group. For perioperative time management, the elapsed time between admission and surgery, the length of operation, and hospital stay were shorter in the ERAS group than in the traditional group. For perioperative nonsurgical management, the ERAS group showed higher rate of regional anesthesia (88.07% *vs*. 49.58%, *P* < 0.001), antiemetic (83.86% *vs*. 73.41%, *P*=0.001), and PCA (85.96% *vs*. 57.5%, *P* < 0.001) than in the traditional group. In the ERAS group, the patients went through less urinary catheterization than in the traditional group (3.86% *vs*. 8.59%, *P* < 0.016). The process of care metrics is shown in Table 3.

### 3.3. Satisfaction Level

The satisfaction levels between the ERAS and the traditional groups were significantly different at discharge and 6 months postoperatively. The patients in the ERAS group had higher satisfaction level than in the traditional group (Table 4).

### 3.4. Multiple Logistic Regression

We defined the satisfaction levels B–D as non-A level and used A and non-A levels as dichotomous dependent variables. When controlling for other demographic, medical, injury, and operative characteristics, age (odds ratio (OR) 0.982, *P*=0.025), regional anesthesia (OR 0.383, *P* < 0.001), PCA (OR 2.816, *P* < 0.001), preoperative fasting period for solids (OR 1.065, *P*=0.024), postoperative fasting period for solids (OR 1.121, *P*=0.004), postoperative fasting period for liquids (OR0.889, *P*=0.006), length of operation (OR0.746, *P*=0.040), and urinary catheterization (OR0.157, *P*=0.001) were identified as significant independent predictors for the satisfaction level of the patients at the time of discharge (Table 5).

### 3.5. Subgroup Analysis

#### 3.5.1. Distal Radial Fracture

Among patients with distal radial fracture, the distribution of the elapsed time between admission and surgery and levels of satisfaction at 6 months postoperatively differed significantly. Patients in the ERAS group had a shorter time between admission and surgery and were more satisfied with the treatment both at discharge and at 6 months postoperatively than those in the traditional group. No difference was noted in the other demographic, medical, injury, and operative characteristics between the two groups (Table 6).

#### 3.5.2. Ankle Fracture

Among patients with ankle fracture, the time in bed (1.0 *vs* 1.4 days, *P* < 0.001) and the length of hospital stay (8 *vs*. 9 days, *P* < 0.001) were significantly shorter in the ERAS group compared to the traditional group, while no difference was observed in the distribution of the satisfaction levels at discharge and 6 months postoperatively between two groups. Also, no difference was detected in the other demographic, medical, injury, and operative characteristics between the two groups (Table 7).

## 4. Discussion

This multicenter prospective cohort study collected the data of 772 patients with ankle fracture across 50 hospitals and 658 patients with distal radial fracture from 41 hospitals and analyzed 323 patients among each population. Pain management, perioperative fasting, and avoiding tubes in the ERAS protocol are the three main constituents. We prescribed multimodal analgesia and minimal fasting time and strived to achieve meticulous hemostasis to reduce wound drains. The results showed satisfactory adherence to the ERAS protocol and demonstrated that the patients in the ERAS group had a higher satisfaction level at discharge and at 6 months postoperatively compared to the traditional group. We also found that age, regional anesthesia, PCA, preoperative fasting period for solids, postoperative fasting period for solids and liquids, the length of operation, and urinary catheterization were independent predictors for the satisfaction level of the patients at the time of discharge. In addition, the ERAS protocol reduced the time in bed and length of hospital stay in ankle fracture patients and elapsed time between admission and surgery in patients with distal radial fracture while maintaining similar early functional outcomes of those treated in the traditional manner and without increasing the rate of complications. These results suggested that the ERAS perioperative protocol was a valuable approach in the management of ankle and distal radial fractures.

Nonetheless, ERAS programs often need a multidisciplinary team that provides a multimodal approach to resolving issues that delay recovery and cause complications based on published evidence. Although the protocol might consist of several elements, one aim is common: minimizing stress and improving the response to stress [1]. ERAS is safe and effective and has been proven to achieve rapid recovery without increased adverse events. The principles are developed best in colorectal surgery [10, 11]. However, the implementation of ERAS programs has also demonstrated satisfying outcomes in many other surgical domains [12–14]. In orthopedic surgery, clinical studies have mainly focused on arthroplasty [15–17]. ERAS principles in orthopedic trauma have begun very late, and the only well-developed ERAS pathway is for elderly hip fractures [8, 9, 18–21]. Kang et al. [9] compared the outcomes of intertrochanteric fracture patients treated in the ERAS pathway to those treated traditionally and found that the protocol reduced the length of hospital stay and preserved hip function without compromising the functional outcomes. Talboys et al. [20] reviewed 100 patients who underwent hemiarthroplasty for a fractured femoral neck retrospectively and concluded that the ERAS program reduces the postoperative oral opiates and PCA requirements. Macfie et al. [21] suggested multimodal optimization as the ERAS principle was associated with a decline in postoperative morbidity in patients with proximal femoral fracture. Due to various surgical options and the high incidence of orthopedic trauma, establishing a scientific, evidence-based ERAS protocol of common fractures is challenging but essential. Therefore, the present study aimed to assess the value of the ERAS principles in common fractures.

This study has several strengths compared to previous studies. First, a large number of patients were enrolled in this study. However, we included 646 patients from different provinces of China, which is a larger sample size than other similar studies [8, 20, 21]. Second, unlike most studies involving ERAS principles in fracture patients, we conducted a prospective study, significantly reducing the risk of information bias. Third, the application of ERAS principles in common fractures other than hip fractures was evaluated for the first time. We included patients with ankle and distal radial fractures as the target population to assess the application of ERAS principles in common fractures because a slight difference was detected between the functional aims and therapeutic strategies of fractures in the upper and lower limbs. Furthermore, the primary outcome of our study was the satisfaction level of the patients at 6 months postoperatively. It is a subjective, patient-oriented evaluation, reflecting the perioperative experience and the short-term functional outcomes of the patients.

Nevertheless, the present study has some limitations. Firstly, the perioperative care was not assigned randomly, which might increase selection bias. Secondly, although there is a standard care protocol for each group, perioperative management may not be homogeneous among different centers because we cannot rule out individualized therapeutic options and measurement errors of some researchers. Thirdly, we only focused on three care elements without assessing the influence of other care elements, such as fluid management and rehabilitation protocol, on patients with common fractures. Lastly, we only evaluated short-term functional outcomes, and additional studies are necessary to assess the long-term effect of the ERAS program on patients with orthopedic trauma.

In conclusion, this study established the perioperative protocol based on the ERAS program with high satisfaction among patients with distal radial fracture and ankle fracture, less time in bed, and short hospital stay without increased complication rate and decreased functional outcomes. Thus, we recommend the ERAS protocol in the management of patients with ankle and distal radial fractures.

## Figures and Tables

**Table 1:** ERAS pathway of ankle and distal radial fractures.

Care process	Description
Preoperative	
Patient education	Verbal counseling and written brochures provided
Oral multimodal analgesia	Oral NSAIDs to prevent hyperalgesia
No prolonged fasting	Clear liquids allowed up to 2 h and solids up to 6 h before anesthesia
Decreased sedative medications	Avoid the use of long-acting anxiolytics the night before surgery
Avoidance of urinary tubes	No routine urinary catheterization
Antibiotic prophylaxis	Antibiotics within 30 min before incision

Intraoperative	
Prevention of hypothermia	Routine body temperature monitoring and active warming devices
Standard anesthesia protocol	Brachial plexus block recommended for distal radial fracture; combined spinal and epidural anesthesia and femoral and/or sciatic nerve block recommended for ankle fracture
Avoidance of drains	Meticulous hemostasis and no wound drains
Fluid management	Avoid too much hypertonic fluid, especially sodium-containing fluid

Postoperative	
Multimodal analgesia	Multimodal opioid-free analgesia based on nerve block
Early feeding	Gradual oral intake of liquid sand solids after recovery from anesthesia
Early mobilization	Early mobilization within 24 h

**Table 2:** Demographic characteristics of the two groups.

	ERAS group	Traditional group	*Z*/*χ*^2^	*P*
Fracture type, Ankle/distal radius (no.)	181/104	142/219	37.227	<0.001
Gender, M/F (no.)	136/149	141/220	4.805	0.028
Age, median (IQR, years)	46.9 (32.9–58.3)	50.3 (38.4–60.5)	2.938	0.003
Injury cause (no.)			23.216	<0.001
Fall from standing height	101	180		
Road accident	28	50		
Falling accident	148	122		
Other causes	8	6		
BMI, median (IQR, kg/m^2^)	24.5 (22.3–26.4)	23.7 (21.5–26.4)	1.689	0.091

BMI, body mass index.

**Table 3:** Process of care metrics of both groups.

	ERAS group	Traditional group	*Z*/*χ*^2^	*P*
Perioperative fasting management				
Preoperative fasting period for solids, median (IQR, h)	15.5 (11.0–19.5)	15.0 (12.1–18.0)	0.125	0.900
Preoperative fasting period for liquids, median (IQR, h)	6.5 (4.1–8.9)	13.8 (11.2–17.0)	19.32	<0.001
Postoperative fasting period for solids, median (IQR, h)	3.0 (2.0–4.7)	5.5 (2.4–8.4)	5.581	<0.001
Postoperative fasting period for liquids, median (IQR, h)	1.5 (0.8–3.2)	4.0 (1.4–6.8)	7.323	<0.001

Perioperative time management				
Elapsed time between admission and surgery, median (IQR, days)	4.0 (3.0–5.0)	4.0 (3.0–6.1)	2.967	0.003
Elapsed time between injury and surgery, median (IQR, days)	5.3 (3.5–7.7)	4.8 (2.9–7.2)	1.873	0.061
Length of operation, median (IQR, h)	1.5 (1.0–1.6)	1.5 (1.2–2.0)	5.479	<0.001
Length of hospital stay, median (IQR, days)	7.0 (6.0–9.0)	8.0 (6.0–13.0)	3.587	0.003

Perioperative nonsurgical management				
Regional anesthesia (no., %)	251 (88.07%)	179 (49.58%)	105.989	<0.001
NSAIDS (no, %)	195 (68.42%)	251 (69.53%)	0.091	0.762
Antiemetic (no., %)	239 (83.86%)	265 (73.41%)	10.146	0.001
Urinary catheterization (no., %)	11 (3.86%)	31 (8.59%)	5.856	0.016
PCA (no., %)	245 (85.96%)	212 (58.73%)	57.094	<0.001
Wound drainage (no., %)	61 (21.40%)	94 (26.04%)	1.876	0.171

NSAIDS, nonsteroidal anti-inflammatory drugs; PCA, patient-controlled analgesia.

**Table 4:** Satisfaction level of both groups.

	ERAS group	Traditional group	*P*
Satisfactory levels on discharge, A : B : C : D (no.)	228/53/4/0	226/129/6/0	<0.001
Satisfactory levels at 6 months postoperatively, A : B : C : D (no.)	154/121/8/2	123/197/41/0	0.001

**Table 5:** Results of multiple logistic regression analysis using patient demographic, injury, and perioperative management characteristics to predict the satisfaction level of the patients on discharge.

Parameter	OR	95% CI	*P* value
Fracture type (distal radial fracture)	1.818	0.956–3.458	0.069
Group (traditional)	1.100	0.553–2.186	0.786
Age	0.982	0.966–0.998	0.025
Gender (female)	1.011	0.644–1.585	0.964
BMI	1.015	0.959–1.074	0.600
Fall from standing height			
Road accident	1.265	0.350–4.577	0.720
Falling accident	0.612	0.157–2.386	0.480
Other causes	1.607	0.419–6.162	0.489
Regional anesthesia	0.383	0.234–0.626	<0.001
PCA	2.816	1.681–4.720	<0.001
NSAIDS	1.041	0.641–1.689	0.872
Preoperative fasting period for solids	1.065	1.008–1.125	0.024
Preoperative fasting period for liquids	0.974	0.912–1.039	0.421
Postoperative fasting period for solids	1.121	1.036–1.213	0.004
Postoperative fasting period for liquids	0.889	0.818–0.967	0.006
Length of operation	0.746	0.563–0.987	0.040
Antiemetic	1.045	0.626–1.743	0.867
Urinary catheterization	0.157	0.053–0.465	0.001
Wound drainage	1.029	0.640–1.654	0.906
Elapsed time between admission and surgery	0.982	0.891–1.081	0.706
Elapsed time between injury and surgery	1.038	0.984–1.096	0.171
Length of hospital stay	1.014	0.950–1.082	0.681

**Table 6:** Subgroup analysis among patients with distal radial fracture.

	ERAS, *n* = 104	Traditional, *n* = 219	*P*
Age, median (IQR, years)	57.0 (48.1–60.9)	55.7 (44.5–62.3)	0.973
Gender, M : F (no.)	42/62	75/144	0.284
BMI, mean ± SD, (kg/m^2^)	24.1 ± 3.2	24.4 ± 3.7	0.448
Fixation method, single volar plate/others (no.)	95/9	196/23	0.603
Blood loss, median (IQR, mL)	20 (16.3–50)	30 (14–60)	0.429
Elapsed time between admission and surgery, median (IQR, days)	4 (3–4)	4 (3–6)	0.001
Length of hospital stay, median (IQR, days)	7 (6–9)	8 (5–12)	0.123
Postoperative complications (no., %)	0 (0)	7 (3.2)	0.065
PRWE, median (IQR)	15 (9–29.8)	16 (11–30)	0.286
Levels of satisfaction on discharge, A : B : C : D (no.)	81/21/2/0	104/110/5/0	<0.001
Levels of satisfaction at 6 months postoperatively, A : B : C : D (no.)	25/74/4/1	20/192/7/0	0.001

PRWE, patient-related wrist evaluation.

**Table 7:** Subgroup analysis among patients with ankle fracture.

	ERAS, *n* = 181	Traditional, *n* = 142	*P*
Age, median (IQR, years)	40.7 (28.9–52.3)	45.1 (33.2–54.3)	0.055
Gender, M : F (no.)	94/87	66/76	0.33
BMI, mean ± SD, (kg/m^2^)	24.6 (22.7–27.0)	24.5 (22.3–27.7)	0.848
Syndesmotic screw, Y/N (no.)	33/148	28/114	0.735
Elapsed time between admission and surgery, median (IQR, days)	4 (3–5)	4 (2.9–7)	0.163
Time in bed, median (IQR, days)	1.0 (0.7–1.9)	1.4 (0.9–2.8)	<0.001
Length of hospital stay, median (IQR, days)	8 (6–10)	9 (6.8–14)	<0.001
Postoperative complications, (no., %)	14 (7.7)	11 (7.7)	0.997
AOFAS ankle hindfoot scale, median (IQR)	99 (89–100)	100 (94–100)	0.228
Levels of satisfaction on discharge, A : B : C : D (no.)	147/32/2/0	122/19/1/0	0.527
Levels of satisfaction at 6 months postoperatively, A : B : C : D (no.)	129/47/4/1	103/5/34/0	0.700

AOFAS, the American Orthopedic Foot and Ankle Society.

## Data Availability

The data and materials could be acquired upon request to the corresponding author via e-mail.
